# Supplementary material for: Phylogenetic and Phylogeographic Analysis of the Highly Pathogenic H5N6 Avian Influenza Virus in China
Source: Viruses. 2022 Aug 11;14(8):1752. doi: 10.3390/v14081752 (PMC9415468; doi:10.3390/v14081752)
Supplement: Supplementary file 1 [file viruses-14-01752-s001.zip › Table S3.pdf]

Table S3. Marginal likelihoods of different combinations of clock model and tree prior.

Fragment	Molecular clock model	Coalescent tree prior	Log marginal likelihood
HA	Strict clock	Bayesian skyline	-7007.357
	Strict clock	Constant size	-7012.430
	Strict clock	Exponential growth	-7007.122
	Uncorrelated lognormal relaxed clock	Bayesian skyline	-6976.358
	Uncorrelated lognormal relaxed clock	Constant size	-6986.254
	**Uncorrelated lognormal relaxed clock**	**Exponential growth**	**-6971.506**

The best-fitting tree prior and molecular clock model are indicated in bold font.
