# Supplementary material for: Phylogenetic and Phylogeographic Analysis of the Highly Pathogenic H5N6 Avian Influenza Virus in China
Source: Viruses. 2022 Aug 11;14(8):1752. doi: 10.3390/v14081752 (PMC9415468; doi:10.3390/v14081752)
Supplement: Supplementary file 1 [file viruses-14-01752-s001.zip › Table S4.pdf]

Table S4. Statistically supported migration rates of the clade 2.3.4.4b and 2.3.4.4h H5N6 AIVs.

Segment	From	To	Bayes_factor [a]	Transition rate[b]	Posterior probability	Inter-province distance (km) [c]
2.3.4.4b HA	Hong Kong	Guangxi	33.77947067	0.969<br>95% HPD [2.6082E-4, 2.9137]	0.784357294	604
	Guangdong	Zhejiang	29.39510454	1.015<br>95% HPD [6.9506E-4, 3.0282]	0.759915565	1048
	Guangdong	Yunnan	23.26441874	0.98<br>95% HPD [3.5402E-5, 2.942]	0.714698367	1092
	Xinjiang	Sichuan	10.64435549	0.977<br>95% HPD [2.8646E-4, 2.8708]	0.534051772	2055
	Hunan	Hong Kong	9.689643112	0.948<br>95% HPD [3.8446E-5, 2.8427]	0.510609932	664
	Sichuan	Xinjiang	9.198651778	0.956<br>95% HPD [2.3529E-5, 2.8898]	0.497611377	2055
	Guizhou	Hunan	4.679946158	0.985<br>95% HPD [5.1355E-5, 2.9192]	0.335073881	645
	Guizhou	Fujian	4.578014832	0.972<br>95% HPD [1.0625E-3, 2.9075]	0.330185535	1255
	Fujian	Guizhou	4.43688389	0.977<br>95% HPD [3.2799E-4, 2.9369]	0.323297411	1255
	Xinjiang	Chongqing	4.380785762	0.98<br>95% HPD [6.1844E-4, 2.8901]	0.320519942	2305

	Hunan	Guizhou	4.059987592	0.938<br>95% HPD [1.2017E-4, 2.7141]	0.304188424	645
	Hong Kong	Guangdong	3.935427321	0.949<br>95% HPD [7.0338E-4, 2.931]	0.297633596	131
	Yunnan	Zhejiang	3.908293767	0.948<br>95% HPD [1.4162E-4, 2.8299]	0.296189312	1811
	Fujian	Hunan	3.839913829	1.246<br>95% HPD [7.6636E-5, 3.2851]	0.292523053	667
	Hong Kong	Chongqing	3.790626435	0.951<br>95% HPD [5.4916E-5, 2.7788]	0.289856683	1107
	Sichuan	Chongqing	3.650964833	1.09<br>95% HPD [2.9583E-4, 2.7848]	0.282190868	270
	Guizhou	Hong Kong	3.63896119	0.944<br>95% HPD [1.2155E-4, 2.9185]	0.281524275	892
	Hong Kong	Sichuan	3.626979799	0.945<br>95% HPD [3.1248E-5, 2.8553]	0.280857682	1366
	Yunnan	Guangdong	3.565428108	0.991<br>95% HPD [1.6741E-3, 2.9161]	0.277413621	1092
	Fujian	Hong Kong	3.35167502	0.998<br>95% HPD [1.9316E-4, 2.9533]	0.265192756	666
	Hunan	Fujian	3.292713733	0.966<br>95% HPD [3.2747E-4, 2.8786]	0.261748695	667

	Hong Kong	Hunan	3.08603167	1.08<br>95% HPD [3.0683E-4, 2.9202]	0.249416731	664
2.3.4.4h HA	Shanxi	Xinjiang	137647.0027	0.985<br>95% HPD [6.9911E-5, 2.9721]	1	2189
	Shanxi	Jiangsu	68815.8543	1.088<br>95% HPD [7.3702E-5, 3.1412]	0.999777802	858
	Shanxi	Guangxi	342.2702959	1.006<br>95% HPD [1.634E-3, 2.8334]	0.957226975	1718
	Anhui	Shandong	283.3225845	0.969<br>95% HPD [1.3538E-3, 2.9199]	0.948783469	534
	Shanxi	Henan	176.436107	1.151<br>95% HPD [3.0579E-4, 3.0305]	0.920231085	357
	Chongqing	Guangdong	154.6593415	0.997<br>95% HPD [1.3918E-3, 2.9171]	0.910009999	976
	Shanxi	Anhui	117.9704547	0.94<br>95% HPD [5.1146E-5, 2.914]	0.885234974	792
	Guangxi	Hunan	59.40056945	1.025<br>95% HPD [4.8296E-3, 2.8584]	0.795244973	758
	Hebei	Hong Kong	37.1886552	0.975<br>95% HPD [3.1931E-4, 3.002]	0.708587935	1748
	Chongqing	Hunan	34.29605315	1<br>95% HPD [1.8592E-3, 2.8886]	0.691589823	642

	Hunan	Jiangxi	31.86666174	1.056<br>95% HPD [4.0394E-4, 2.9998]	0.6757027	290
	Shanxi	Hebei	23.92591617	0.97<br>95% HPD [4.6956E-4, 2.9521]	0.610043329	168
	Anhui	Guizhou	21.92197316	0.977<br>95% HPD [1.1317E-3, 2.9991]	0.589045662	1181
	Hebei	Guangdong	20.65843412	0.995<br>95% HPD [1.8693E-5, 2.8415]	0.574602822	1660
	Xinjiang	Sichuan	15.19587681	0.959<br>95% HPD [3.5149E-4, 2.8922]	0.498389068	2057
	Hong Kong	Fujian	11.76747633	0.974<br>95% HPD [1.6882E-4, 2.9745]	0.434840573	667
	Hebei	Chongqing	8.886864668	0.979<br>95% HPD [5.9426E-4, 2.9928]	0.367514721	1190
	Shanxi	Chongqing	7.959654943	1.019<br>95% HPD [1.7912E-4, 2.9217]	0.342295301	1073
	Chongqing	Fujian	7.62668393	1.02<br>95% HPD [5.2197E-4, 2.881]	0.332740807	1308
	Chongqing	Hebei	6.108608865	0.99<br>95% HPD [2.7566E-3, 2.8778]	0.285412732	1190
	Hebei	Fujian	4.962259515	0.935<br>95% HPD [6.0006E-4, 2.9057]	0.244972781	1404

	Fujian	Shanxi	4.899859077	0.996<br>95% HPD [1.3933E-3, 2.8821]	0.242639707	1453
	Fujian	Hunan	4.787915841	0.961<br>95% HPD [7.3994E-5, 2.8888]	0.238417954	667
	Shanxi	Shandong	4.714943359	0.994<br>95% HPD [2.2019E-4, 2.9326]	0.235640484	415
	Xinjiang	Chongqing	4.61654766	0.98<br>95% HPD [9.7682E-4, 2.9813]	0.231863126	2306
	Hebei	Shandong	4.307525612	0.941<br>95% HPD [3.1327E-5, 2.8385]	0.219753361	272
	Xinjiang	Hebei	4.268528466	0.997<br>95% HPD [1.9588E-4, 2.9368]	0.218197978	2332
	Xinjiang	Hainan	3.919049345	0.924<br>95% HPD [3.0884E-3, 2.8984]	0.203977336	3501
	Chongqing	Shandong	3.654587393	0.977<br>95% HPD [2.459E-3, 3.0709]	0.192867459	1251
	Jiangsu	Shanxi	3.326515779	1.087<br>95% HPD [3.5184E-5, 2.6818]	0.178646817	858
	Xinjiang	Guangxi	3.221328645	0.981<br>95% HPD [2.871E-4, 2.9762]	0.173980669	3006
	Jiangsu	Hainan	3.019350983	0.993<br>95% HPD [3.5278E-4, 2.8047]	0.16487057	1794

	Hong Kong	Hebei	3.014479733	0.995<br>95% HPD [4.5469E-5, 2.9519]	0.164648372	1748
	Shanxi	Guizhou	3.004745005	0.968<br>95% HPD [3.5259E-6, 2.9814]	0.164203977	1367
	Guangxi	Jiangxi	3.004745005	1.01<br>95% HPD [3.3876E-4, 2.8935]	0.164203977	999

[a] Only statistically supported migrations with Bayes factor >3 are shown.

[b] 95% highest probability density (HPD).

[c] The inter-province distance is represented by the inter-provincial capital city distance, obtained from https://ditu.google.com.
