# Supplementary figures and images for: Phylogenetic and Phylogeographic Analysis of the Highly Pathogenic H5N6 Avian Influenza Virus in China
Source: Viruses. 2022 Aug 11;14(8):1752. doi: 10.3390/v14081752 (PMC9415468; doi:10.3390/v14081752)

# Figure S1

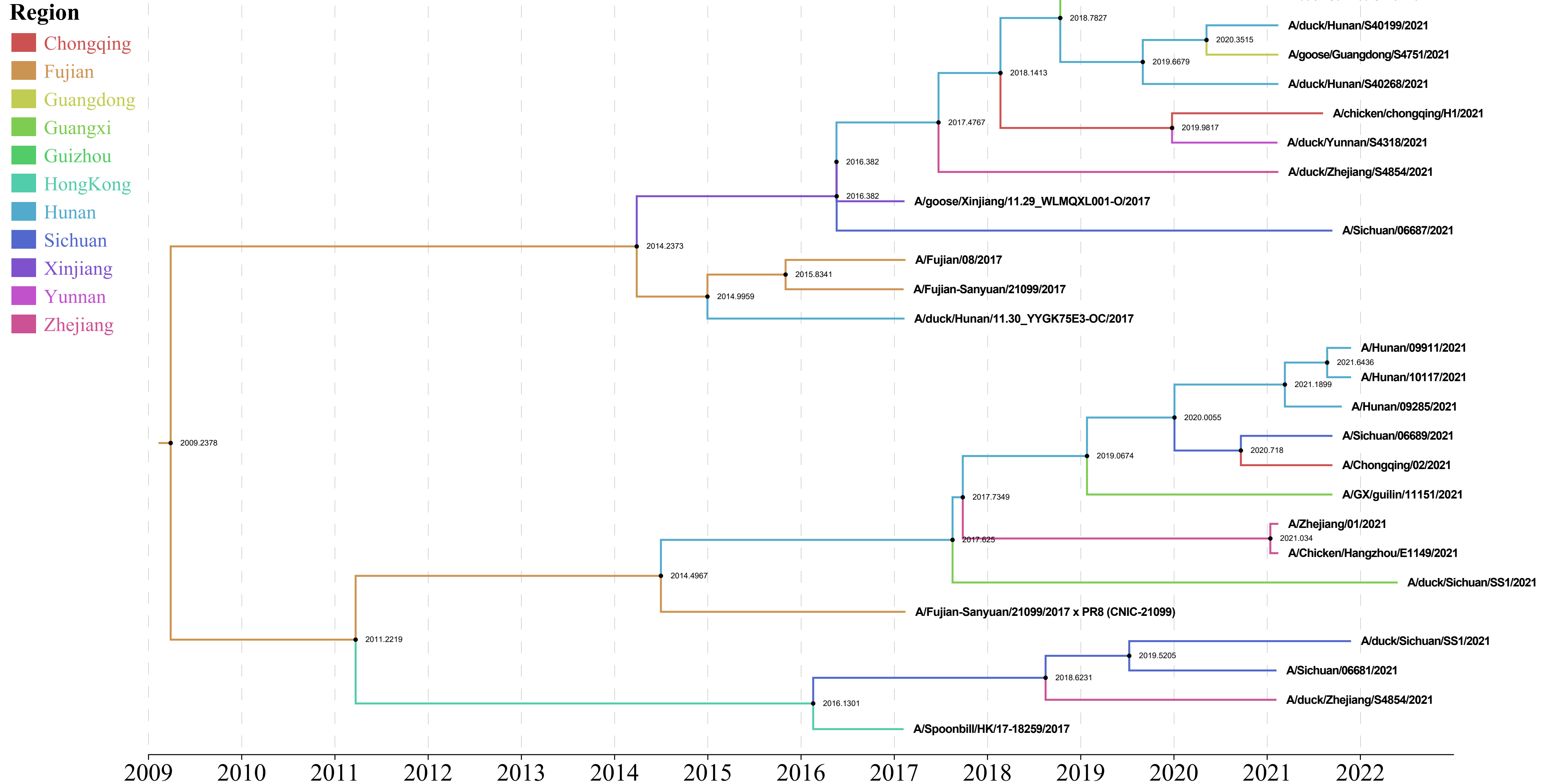

Supplement: Supplementary file 1 [file viruses-14-01752-s001.zip › Figure S1 2.3.4.4b MCC.pdf]

NS

Colored ranges

H5N1

H5N8

H3N2

H7N9/H9N2

Other subtypes

Tree scale: 0.01

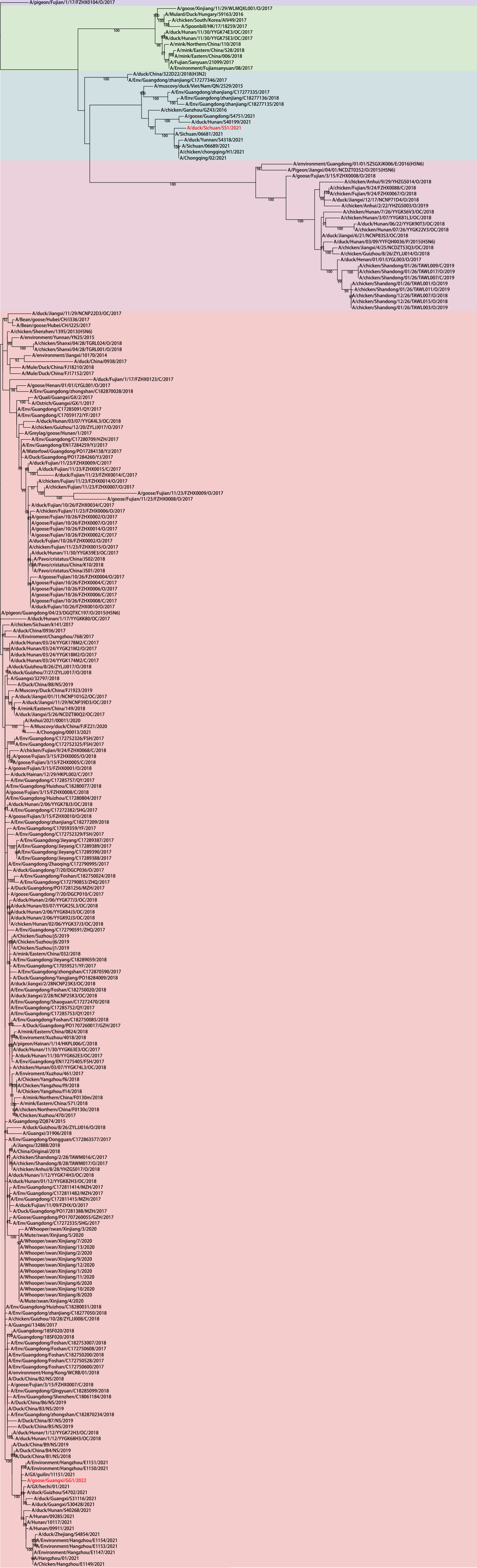

Supplement: Supplementary file 1 [file viruses-14-01752-s001.zip › Figure S10 NS-tree.pdf]

Figure S2

Region

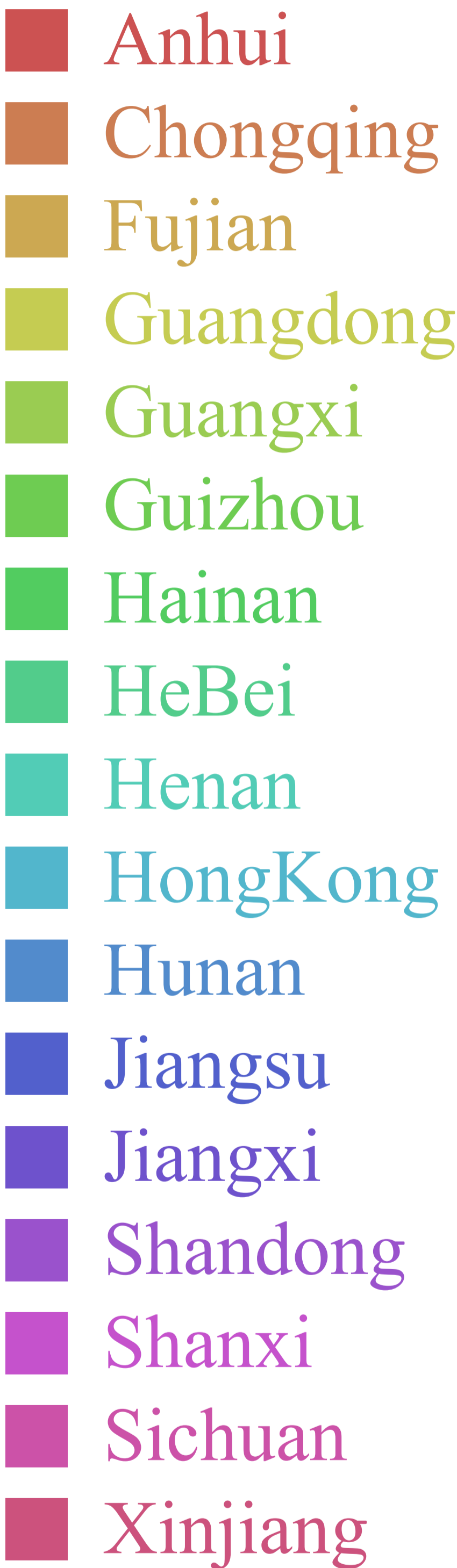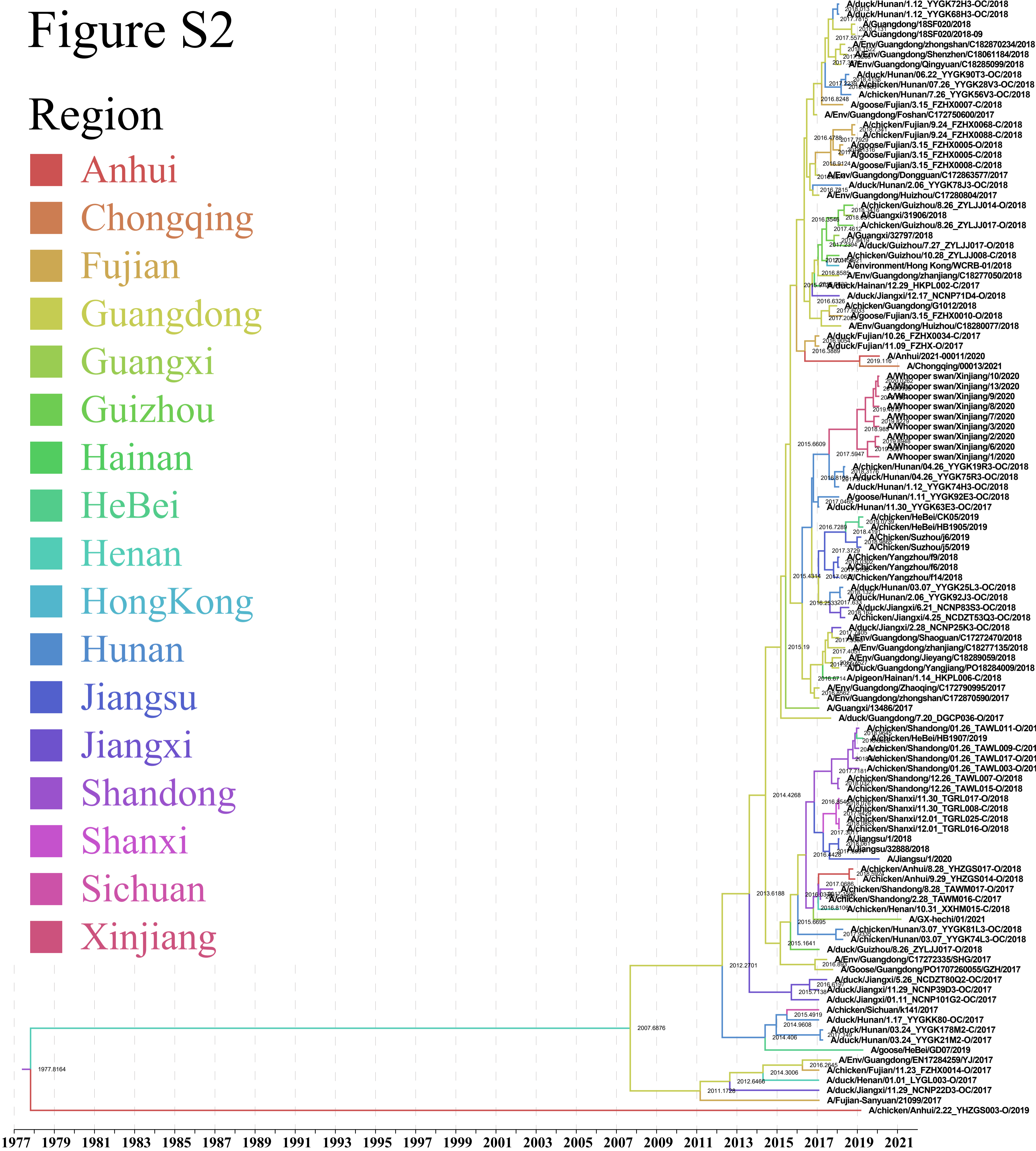

Supplement: Supplementary file 1 [file viruses-14-01752-s001.zip › Figure S2 2.3.4.4h MCC.pdf]

HA

Clade

- 2.3.4.4c
- 2.3.4.4b
- 2.3.4.4g
- 2.3.4.4e
- 2.3.4.4h

Tree scale: 0.01

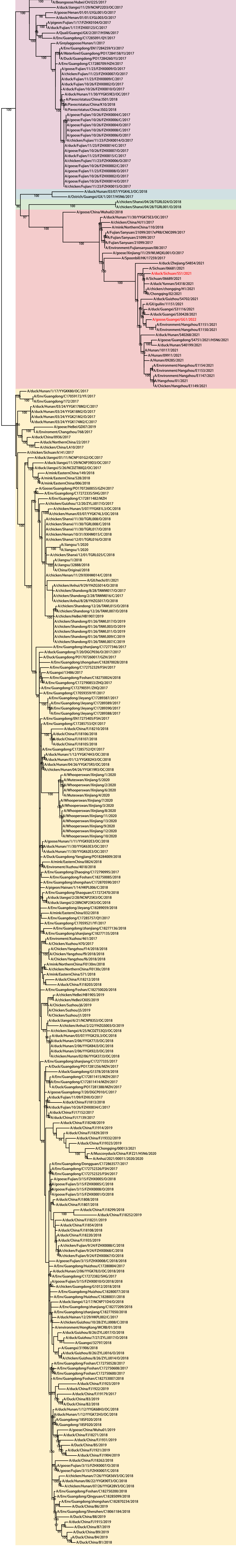

Supplement: Supplementary file 1 [file viruses-14-01752-s001.zip › Figure S3 HA-tree.pdf]

## Lineage

## Lineage

- North-American
- Europe
- Eurasian

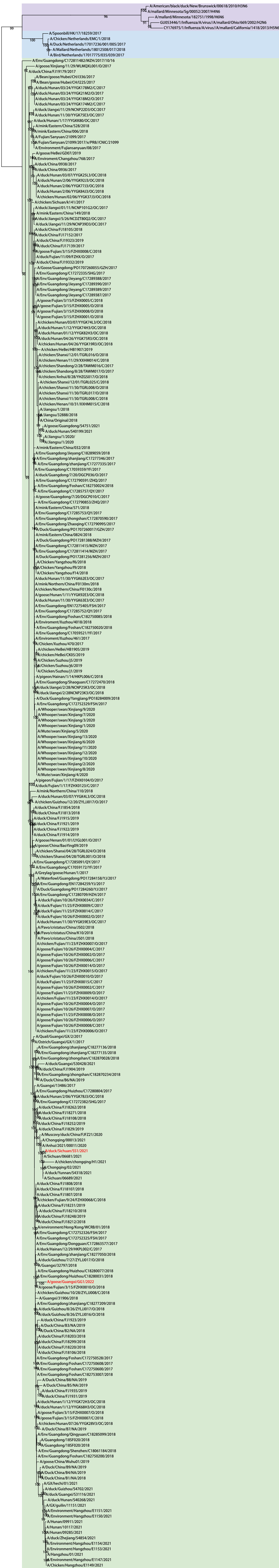

Supplement: Supplementary file 1 [file viruses-14-01752-s001.zip › Figure S4 NA-tree.pdf]

PB2

Colored ranges

- H3N2
- H5N8
- H5N1
- H7N9/H9N2
- H6

Tree scale: 0.01

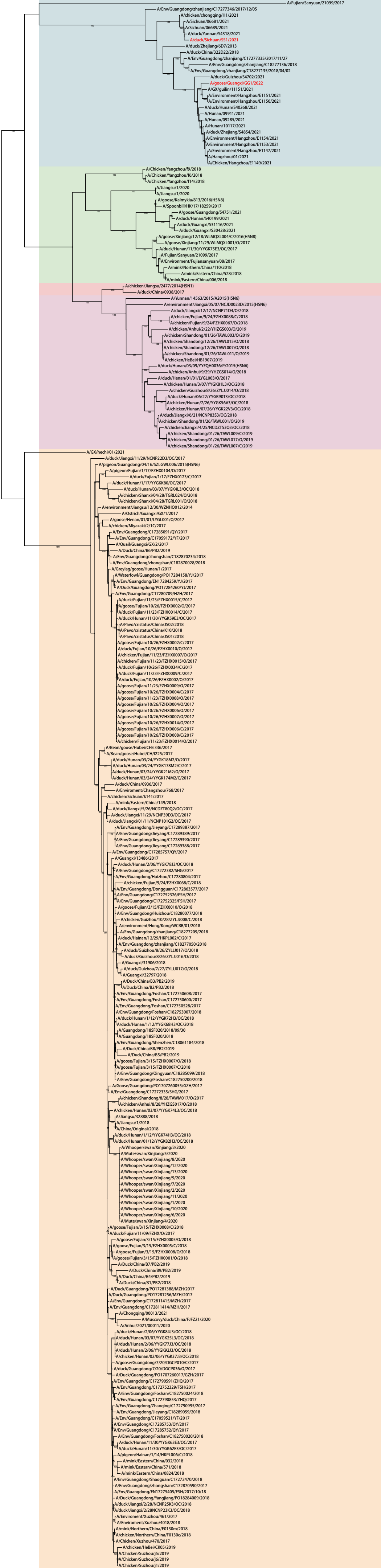

Supplement: Supplementary file 1 [file viruses-14-01752-s001.zip › Figure S5 PB2-tree.pdf]

# PB1

## Colored ranges

- H7N9/H9N2
- H5N8
- H5N6
- H5N1
- H3N2

Tree scale: 0.01

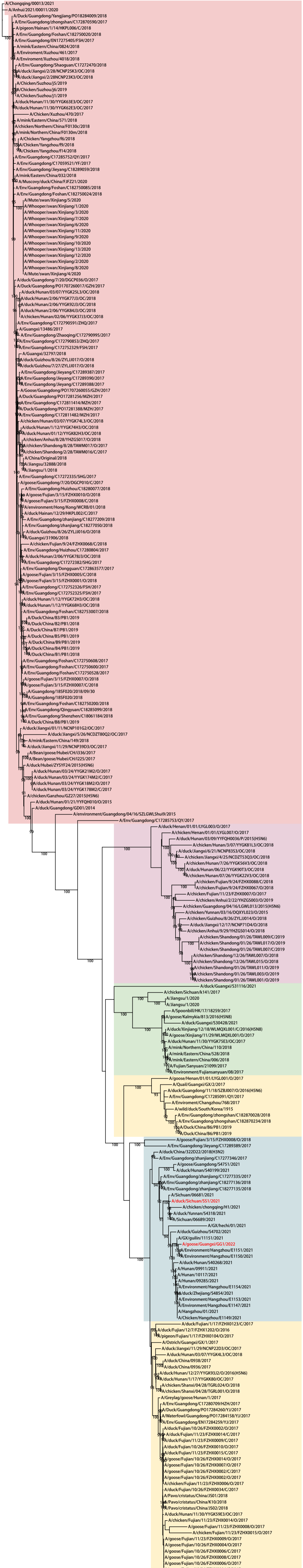

Supplement: Supplementary file 1 [file viruses-14-01752-s001.zip › Figure S6 PB1-tree.pdf]

PA

Colored ranges

- H3N2
- H7N9/H9N2
- H5N6
- H5N8
- H5N1
- Other subtypes

Tree scale: 0.01

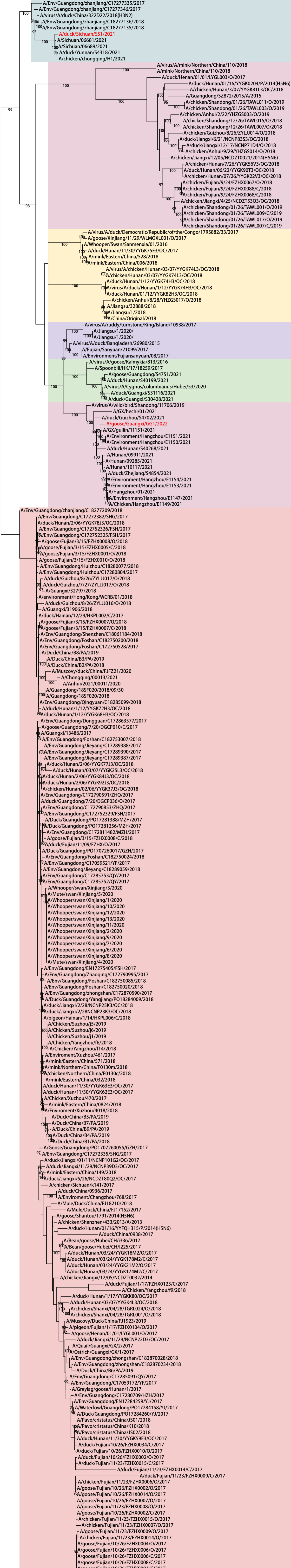

Supplement: Supplementary file 1 [file viruses-14-01752-s001.zip › Figure S7 PA-tree.pdf]

# NP

## Colored ranges

- H7N9/H9N2
- H3N2
- H5N8
- H5N1

Tree scale: 0.01

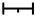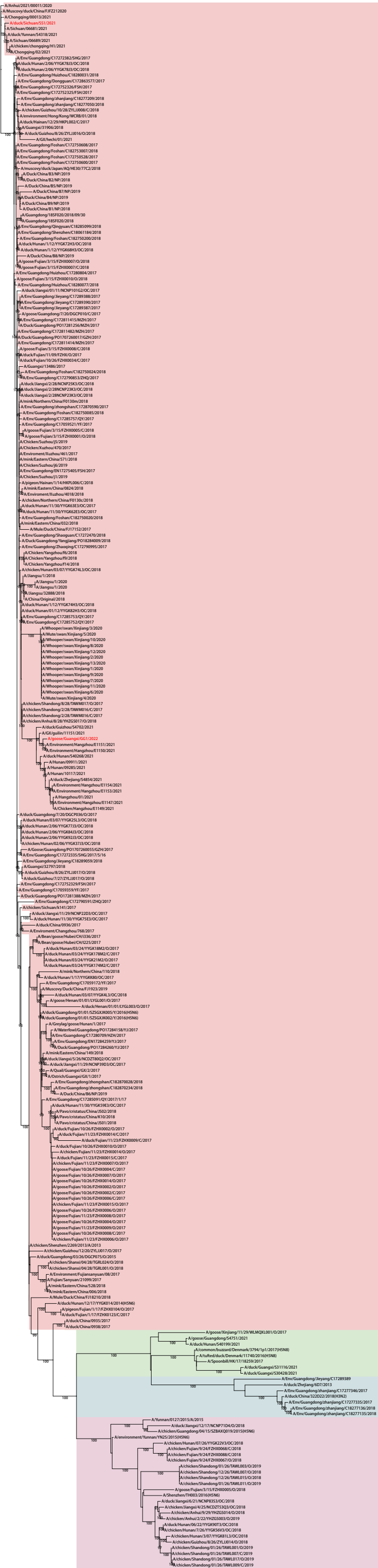

Supplement: Supplementary file 1 [file viruses-14-01752-s001.zip › Figure S8 NP-tree.pdf]

M

Colored ranges

- H3N2
- H7N9/H9N2
- H5N1
- H5N6
- H5N8

Tree scale: 0.01

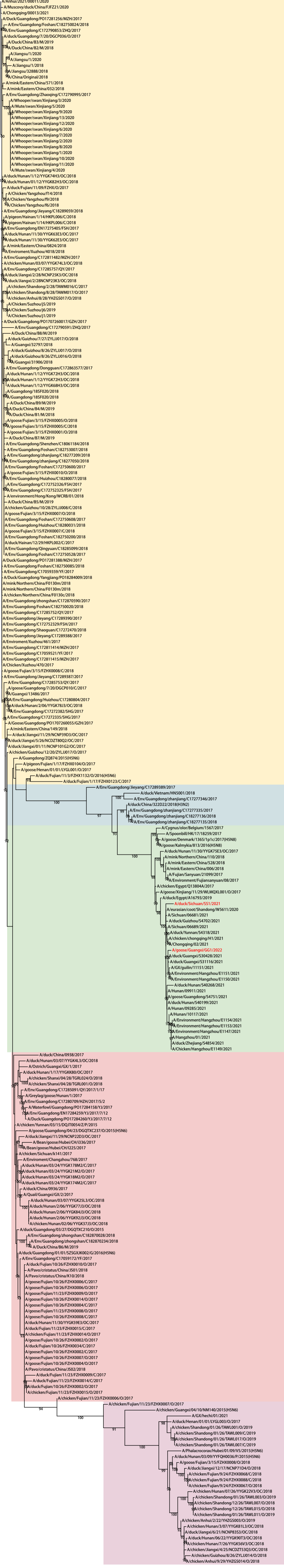

Supplement: Supplementary file 1 [file viruses-14-01752-s001.zip › Figure S9 M-tree.pdf]
